# Disruption of Functional Brain Networks Underlies the Handwriting Deficit in Children With Developmental Dyslexia

**DOI:** 10.3389/fnins.2022.919440

**Published:** 2022-07-18

**Authors:** Zhengyan Liu, Junjun Li, Hong-Yan Bi, Min Xu, Yang Yang

**Affiliations:** ^1^CAS Key Laboratory of Behavioral Science, Center for Brain Science and Learning Difficulties, Institute of Psychology, Chinese Academy of Sciences, Beijing, China; ^2^Department of Psychology, University of Chinese Academy of Sciences, Beijing, China; ^3^Center for Brain Disorders and Cognitive Sciences, School of Psychology, Shenzhen University, Shenzhen, China

**Keywords:** developmental dyslexia, handwriting, fMRI, functional brain network, children

## Abstract

Developmental dyslexia (DD) is a neurological-based learning disorder that affects 5-17.5% of children. Handwriting difficulty is a prevailing symptom of dyslexia, but its neural mechanisms remain elusive. Using functional magnetic resonance imaging (fMRI), this study examined functional brain networks associated with handwriting in a copying task in Chinese children with DD (*n* = 17) and age-matched children (*n* = 36). We found that dyslexics showed reduced network connectivity between the sensory-motor network (SMN) and the visual network (VN), and between the default mode network (DMN) and the ventral attention network (VAN) during handwriting, but not during drawing geometric figures. Moreover, the connectivity strength of the networks showing group differences was correlated with handwriting speed, reading and working memory, suggesting that the handwriting deficit in DD is linked with disruption of a large-scale brain network supporting motoric, linguistic and executive control processes. Taken together, this study demonstrates the alternations of functional brain networks that underly the handwriting deficit in Chinese dyslexia, providing a new clue for the neural basis of DD.

## Introduction

Developmental Dyslexia (DD) is a learning disorder that is characterized by unexpected reading difficulties despite adequate intelligence and educational opportunities. It affects 5-17.5% of children (Shaywitz, 1998). Previous neuroimaging studies have demonstrated that dyslexia is associated with the abnormalities of regional activity and functional connectivity in multiple brain systems including the left hemisphere reading network (e.g., the temporoparietal cortex, inferior frontal gyrus and occipitotemporal cortex) (Shaywitz et al., 2002; Hoeft et al., 2007; van der Mark et al., 2009, 2011; Boets et al., 2013; Olulade et al., 2013; Finn et al., 2014) and the cerebellum (Nicolson et al., 1999; Menghini et al., 2006; Yang et al., 2013).

In addition to reading difficulties, dyslexics pervasively exhibit handwriting deficits (Graham et al., 2021). For example, children with DD show poorer writing legibility (Martlew, 1992) and larger size of written scripts than typically developed children (Lam et al., 2011). Moreover, relative to typical readers, dyslexic readers were found to show increased writing latency (Afonso et al., 2020), reduced motor speed (Pagliarini et al., 2015) and more pauses (Sumner et al., 2012, 2014) during handwriting. Chinese is a logographic/morphosyllabic writing system, in which a grapheme corresponds to a syllabic morpheme (Perfetti and Harris, 2013). There is a lack of one-to-one correspondence between phonology and orthography in Chinese, and what’s more, there are many homophonic characters. At the script level, character is the basic written unit in Chinese that has a square configuration consisting of many radicals and strokes, resulting in a high level of visual complexity. Due to the linguistic and visual features, handwriting becomes a prevalent strategy for masting Chinese reading via the elaboration of orthographic representation and the formation of motor memory (Tan et al., 2005, 2013). Accordingly, the handwriting problem is expected to be more relevant with dyslexia in Chinese than that in alphabetic languages (e.g., English) (Kalindi et al., 2015).

Handwriting is a complex process involving linguistic, motor and executive control processes, and thus the underlying causes of the handwriting deficit in DD is likely multifactorial. One possibility is that the handwriting deficit is derived from orthographic processing impairments in dyslexics (Cao et al., 2006; Boros et al., 2016), which lead to an inability to extract and to operate orthographic information quickly and accurately during handwriting. This hypothesis is supported by existing empirical evidence showing that dyslexics are more impacted by orthographic complexity (Arfe et al., 2020) or spelling regularity (Sumner et al., 2014) than typical readers during handwriting. Another possibility is that motor skill impairment is the origin of the handwriting deficit in DD. For instance, kinematic measures of handwriting processing revealed that, relative to typically developed readers, dyslexics showed increased motor variability (Pagliarini et al., 2015) and greater vulnerability to motor complexity (Gosse and Van Reybroeck, 2020) in handwriting.

Despite the extensive research on behavioral manifestations, the neural bases of the handwriting deficit in DD remain largely unknown. An fMRI study has reported that French-speaking dyslexic children showed reduced brain activation in the right anterior cerebellum relative to age-matched typically developed children in a dictation task (Gosse et al., 2022). The cerebellum is a key brain locus of motor processing, and thus this result favors the motor impairment hypothesis. However, this study used a region of interest (ROI) analysis approach, which can hardly delineate the full map of brain dysfunction associated with the handwriting deficit in DD. Recently, another fMRI study investigated the neural basis of the handwriting deficit in Chinese dyslexic children. Whole brain analysis revealed that Chinese dyslexic children showed decreased brain activation in somatomotor regions (the supplementary motor area (SMA) and postcentral gyrus) and visual-orthographic regions (the bilateral precuneus and right cuneus), while showed hyperactivation in the left inferior frontal gyrus and anterior cingulate cortex. Moreover, using seed-to-voxel connectivity analysis, this study revealed aberrant functional connectivity within the neural circuits for cognitive control and sensory-motor processes involved in handwriting in dyslexics (Yang et al., 2022). These findings suggest that the handwriting difficulty in DD is linked with a malfunction of distributed brain systems involved in motor, visual-orthographic and executive control processes. However, local activity and seed-based functional connectivity analyses are still not sufficient to decipher the large-scale interaction between brain regions involved in handwriting.

According to the graph theory of brain, functional and structural brain systems are organized as graphs formed by highly connected hubs and modularity (Bullmore and Sporns, 2009). Brain network analysis provides an intuitive and powerful way for illustrating the topological principles of brain function underlying complex cognitive processes and neurological disorders (Sporns, 2011). Such network analysis method has been applied in the investigation of the neural signatures of dyslexia, revealing that dyslexia is related to altered functional connectivity in multiple brain networks during rest (Finn et al., 2014) and task states (Zhang J. et al., 2021).

This study used a network analysis approach to explore the alternations of functional brain networks underlying the handwriting deficit in Chinese children with dyslexia. A delayed copying task was used, which is thought to have the advantages in controlling high-order linguistic/cognitive processes (Yang et al., 2022) Furthermore, to examine the influence of linguistic factors to motor execution in handwriting (Kandel and Perret, 2015), the frequency of character was manipulated in this study. Character frequency is a lexical variable that has been found to impact orthographic access during handwriting (Yang et al., 2018). We hypothesized that dyslexics would show disruption in multiple functional networks related to handwriting including the sensorimotor network, visual network and cognitive control network. Moreover, functional disruption of the motor and visual networks in DD was expected to be independent of character frequency, whereas group differences in cognitive control networks are mediated by character frequency.

## Materials and Methods

### Participants

Seventeen children with DD (11 males) and 36 age-matched controls (15 males) participated in this study. The dyslexic participants were screened according to the following criteria: (1) the score of the Character Recognition Measures and Assessment Scale (CRM) (Wang, 1986) was at least 1.25 standard deviations (SD) below the average score of children in the same grade. The CRM is a standardized reading test that has been widely used for screening dyslexia in Mandarin-speaking children (Amalric and Dehaene, 2016; Zhang et al., 2018; Feng et al., 2020; Yang et al., 2022); (2) having a normal non-verbal intelligence quotient (IQ) standardized score (above 85) as evaluated by Combined Raven’s Progressive Matrices; (3) having normal hearing, normal or corrected-to-normal vision, and no ophthalmological or neurological abnormalities; and (4) not suffering from attention deficit/hyperactivity disorder (ADHD) evaluated by the Chinese Classification of Mental Disorder 3 (CCMD-3). All the children were native speakers of Mandarin, and were right-handed as assessed by the Handedness Inventory (Snyder and Harris, 1993). The study was approved by the ethics committee of the Institute of Psychology at the Chinese Academy of Sciences. Prior to entering the study, written informed consent was obtained from the guardian of each child participant. Detailed participant information was listed in Table 1.

**TABLE 1:** Demographic information of the participants and behavioral performance.

	Dyslexics (*n* = 17)	Controls (*n* = 36)	*P*-value
Sex (male/female)	11/6	15/21	0.117
Age	10.28 (0.57)	10.40 (0.54)	0.479
Raven IQ	105.76 (9.43)	111.81 (15.56)	0.087
CRM	1978.1 (315.65)	2908.46 (261.6)	<0.001
**Reading-related skills**			
Reading fluency (character/min)	65.29 (19.06)	100.58 (19.57)	<0.001
Phonological awareness	25.24 (3.99)	28.11 (2.04)	0.001
Orthographic awareness			
Mean ACC	0.75 (0.14)	0.84 (0.08)	0.004
Mean RT (in ms)	994.41 (143.2)	865.09 (137.46)	0.003
**Handwriting skill**			
Copying tasks			
Speed (characters/s)	0.46 (0.09)	0.50 (0.10)	0.226
Quality	24.75 (4.24)	25.55 (6.07)	0.628
Handwriting fluency			
Characters	24.35 (6.24)	27.64 (4.82)	0.040
Digits	51.88 (8.91)	58.50 (12.20)	0.051
**Cognitive skill**			
Phonological working memory	4.76 (1.03)	6.33 (1.64)	<0.001
Sustained attention	29.06 (7.84)	31.59 (5.98)	0.253

*IQ = intelligence quotient, CRM = the Character Recognition Measures and Assessment Scale, ACC = accuracy, RT = response time, ms = millisecond, min = minute and s = second.*

### Behavioral Tests

A series of behavioral tests were administered to examine reading, handwriting and domain-general cognitive skills of the participants.

#### Reading Skills Tests

Reading-related skills including reading fluency, phonological awareness and orthographic awareness were assessed. The reading fluency test consisted of 160 Chinese characters of medium to high frequency. The participants were asked to read aloud these characters as fast and accurately as possible within 1 min. The number of correctly named characters was defined as the final score. Phonological awareness was assessed by using the oddity tests. In this test, the participants were required to listen carefully to three syllables, and were then asked to orally report the odd syllable that differed in initial sound, final sound, or tone with the other two syllables. The final score was defined as the total number of items correctly answered. There were 10 items for each type of stimuli, and thus the maximum score was 30. Finally, orthographic awareness was evaluated in a character judgment task. This test consists of 40 real Chinese characters, 20 pseudo-characters and 20 non-characters. Participants were asked to judge whether the stimuli were real Chinese characters or not. The mean accuracy (ACC) and reaction time (RT) of real characters, pseudo-characters and non-characters were defined as the final score.

#### Handwriting Skills Tests

Handwriting skills were assessed in a copying task and a handwriting fluency task. In the copying task, participants were required to copy 48 Chinese characters using habitual writing styles (Yang et al., 2022). Writing quality and speed were evaluated. Writing quality was evaluated by two independent (one male) examiners using a 7-point Likert scale (1 = very bad and 7 = very good) based on six dimensions, including stroke form, slant, organization of radicals, neatness, average size, and overall appearance (Gimenez et al., 2014; Yang et al., 2020). The score was the sum of the sub-scores across all dimensions. The inter-rater reliability of the assessment was high (intra-class correlation coefficient (ICC) = 0.92). In the handwriting fluency test, participants were asked to continuously handwrite a Chinese sentence 妈妈永远爱我 (“Mommy loves me for forever”) or digits ranging from 1 to 10 in Chinese as fast and as legibly as possible within 1 min. The score was the number of legible characters or digits.

#### Cognitive Skills Tests

Working memory and sustained attention were assessed because they are necessarily involved in handwriting and reading processes. First, phonological working memory was measured by using a backward digit span task, in which participants were asked to orally reproduce digits (3 to 10 digits) in the reverse order as they were presented (Zhang et al., 2018; Yang et al., 2022). The test was terminated when the participants failed in two consecutive trials of the same length, and the score was the maximum length of digit span with a correct response. Second, sustained attention was assessed using a digit cancellation test (Yang et al., 2022). Participants were required to search the target number (“3”) from a list of numbers as quickly and accurately as possible within 3 min. The score was calculated according to the following formulas: score = attack - (false alarms + 0.5*omission), where attack was the number of correctly marked items, false alarms was the number of incorrectly marked items, and omission was the number of items missed.

### Stimuli and Task Procedure During Functional Magnetic Resonance Imaging

The participants performed a delayed copying task during fMRI scanning. The stimuli included thirty-two Chinese characters, including 16 high-frequency characters (HFCs) (mean frequency = 2486 times per million) and 16 low-frequency characters (LFCs) (mean frequency = 91 times per million), according to the Wang (1986). In addition, a drawing condition was included as a control condition for excluding low-level visual and motor processes, in which participants were asked to draw geometric figures (line, dot, circle, and triangle) as instructed by presentation of the appropriate Chinese characters. A direct copying task was also included as part of a large study. However, as this condition rarely taps the processing of orthographic working memory that we were interested in, it was not analyzed in the present study. Participants were instructed to start handwritten or drawn responses when the cursor appeared (a pencil symbol).

Handwriting data were recorded using a tablet system that includes a touch-sensitive surface, a force-sensitive stylus and an adjustable support frame, which is MRI-safe without significantly degrading fMRI data quality (Tam et al., 2011). Participants used the stylus to write on the surface. The support frame was adjusted carefully for each participant to ensure that handwriting and drawing could be undertaken comfortably throughout the imaging session, and to enable tablet interaction with the forearm or wrist resting on the support such that there was no fatigue from handwriting against gravity. To approximate real handwriting, immediate visual (“ink”) feedback was provided via a mirror installed in the scanner that can reflect the writing traces displayed on the computer screen during writing responses. Participants were trained to write and draw with matched duration and size, while minimizing movements of their upper arm and forearm to minimize task-related head motion during fMRI scanning.

A block design was employed in this study, with four blocks for each condition. Each block consisted of an instruction presenting for 2 s and subsequent four trials. In each trial, a fixation cross (‘+’) was first presented centrally for 0.5 s, followed by the presentation of a character for 1.2 s. Then, a blank screen was displayed during a delay period of 0.5 s; afterwards, the cursor appeared to allow participants to write or draw within the response period of 5.3 s. Eight blocks of central fixation with 12 s duration were interspersed between each of the two task blocks as a “rest” condition. Each participant underwent two fMRI runs, and each run consisted of two blocks of task condition and 8 rest blocks. Detailed information about the experimental design and fMRI scanning procedures have been reported previously (Yang et al., 2022).

### Imaging Acquisition

Imaging was performed using a 3T MRI system (MAGNETOM Prismafit, Siemens, Erlangen, Germany) at the Beijing MRI Center for Brain Research of the Chinese Academy of Sciences. Functional MRI time series data with blood oxygenation level-dependent (BOLD) contrast were acquired using a two-dimensional, T2*-weighted, multiband gradient-echo echo planar imaging sequence (Moeller et al., 2010): four-fold acceleration, repetition time (TR) = 1000 ms, echo time (TE) = 30 ms, slice thickness = 2.2 mm, in-plane resolution = 2.2 × 2.2 mm, flip angle (θ) = 45°, 64 axial slices. High spatial resolution anatomical images were acquired using a three-dimensional, T1-weighted, magnetization-prepared rapid acquisition gradient echo sequence: TR = 2200 ms, TE = 3.49 ms, slice thickness = 1 mm, inversion time (TI) = 1000 ms, in-plane resolution = 1.0 × 1.0 mm, and θ = 8°.

### Data Analysis

#### Behavior Data

Handwriting latency and duration were analyzed for the delayed copying task and the drawing task during fMRI scanning. Writing latency was defined as the time period between the appearance of the response screen and the start of the response (first contact with the tablet), while writing duration was defined as the length of time from the start of the response to the end of the last written or drawn stroke of the response. A 2 (group: dyslexics vs. controls) by 3 (stimulus type: HFC vs. LFC vs. figures) analysis of variance (ANOVA) was conducted for writing latency and duration, respectively. The statistical significance was set at *p* < 0.05.

### Image Data

#### Preprocessing

Image preprocessing and statistical analyses were performed using SPM12 freeware (^1^Wellcome Department of Cognitive Neurology, University College London, London). The fMRI time series data for each participant were first corrected for head motion, and the corrected images were coregistered to the associated anatomical imaging data. The anatomical images were then transformed into Montreal Neurological Institute (MNI) stereotactic space, and the resulting transformation parameters were applied to yield fMRI time series data normalized in MNI space with cubic voxels at a spatial resolution of 2 mm × 2 mm × 2 mm. These images were then spatially smoothed using an isotropic Gaussian kernel template with 6 mm full-width at half-maximum. Three dyslexic children were excluded from the data analysis because of excessive head motion (>3 mm translation or >3° rotation) during fMRI scanning, and a dyslexic child was excluded due to poor quality of T1-weighted images. For age-matched controls, six children were excluded because of excessive head motion (>3 mm translation or >3° rotation) during fMRI scanning, and seven controls were excluded due to poor quality of T1-weighted images. Accordingly, thirteen children with dyslexia and twenty-three age-matched controls were included in further statistical analysis. The head motion was quantified by calculating the mean framewise displacement (FD) (Power et al., 2012) based on the estimates of the six head movement parameters. Independent two-sample *t*-tests indicated that dyslexics and controls did not differ in FD (*t*(34) = −0.08, *p* = 0.933).

#### Creation of Functional Connectivity Matrices

Functional connectivity (FC) matrices were created using the CONN functional connectivity toolbox (Whitfield-Gabrieli and Nieto-Castanon, 2012). First, 264 ROIs in spheres with 10-mm diameter were defined as nodes based on a validated parcellation template (Power et al., 2011; Cole et al., 2013). Then, BOLD time series signals corresponding to the conditions of HFC, LFC and figure were separately extracted from each ROI, and were then concatenated over blocks. Nuisance BOLD signal fluctuations from cerebrospinal fluid and white matter were estimated and removed using the anatomical component correction (CompCor) strategy (Behzadi et al., 2007). In addition, head motion (Six motion parameters and six first-order temporal derivatives) as well as the main effect of task were also regressed out. The data were high-pass filtered at 0.008 Hz to preserve task-relevant high-frequency signals. Next, Pearson’s correlation coefficients between each pair of regional time series signals were computed and transformed into Fisher’s *z* scores. Following this procedure, undirected and weighted 264 × 264 FC matrices were constructed for each condition and for each participant. Finally, the significant non-zero connections in FC matrices were defined as the significant edges for each condition by performing one-sample *t*-tests (false discovery rate (FDR) corrected *p* < 0.05) using the GRETNA toolbox (Wang et al., 2015).

#### Network-Based Statistical Analysis

The network-based statistic (NBS) analysis was applied to identify differences in the functional networks involved in handwriting and drawing between dyslexics and controls. NBS is a non-parametric method that can detect the specific connections within brain networks for the differences between psychological contexts. This approach rejects the null hypothesis on a component-basis controlling for family-wise error (FWE) rate, and thus achieves substantially greater statistical power than mass-univariate testing performed at the edge level (Zalesky et al., 2010). At the group level, independent two-sample *t* tests were applied for the HFC, LFC and figure condition respectively, because we were particularly interested in the group differences in functional network reconfiguration during handwriting. Factors including sex, age, Raven IQ and FD were included as covariates to account for the potential confounding effect. In addition, to account for the differences in behavioral performance during fMRI scanning, writing duration and writing latency during fMRI were also included as covariates. A mask containing the significant edges across the groups and conditions was applied to the group analysis, ensuring that statistical comparisons were restricted within a same network space (Jiang et al., 2013). A set of supra-threshold connections were first defined (*p* < 0.01), which was used to determine the topological components and their intensity-based sizes (the sum of test statistic values across all connections within a component) (Zalesky et al., 2012; Cao Q. et al., 2013; Spies et al., 2019). Then, non-parametric permutation tests were performed to estimate the significance of each component (permutation times = 5000, family-wise error (FWE) rate corrected *p* < 0.05). For each permutation, the labels of participants were randomized under the null hypothesis without affecting the test statistic (Zalesky et al., 2012). Finally, the corrected *p* value for a component of a given size was calculated as the proportion of permutations for which the largest component was the same size or greater. To further specify the function of networks, the identified networks were assigned to a well-established brain network partition consisting of 10 well-defined brain systems (frontoparietal, cingulo-opercular, default mode, dorsal attention, ventral attention, auditory, visual, salience, somatomotor and subcortical networks) (Power et al., 2011). Given an edge connects two nodes belonging to a same network, this edge was defined as a within-network functional connectivity. While, when an edge connects two nodes belonging to two different networks, this edge was defined as between-network functional connectivity.

In addition, the hubs were defined as the nodes with a connectivity strength of SD greater than the mean strength across all nodes in the network (Sporns et al., 2007; Liu et al., 2018). Node strength is analogous to node degree in weighted networks, which is defined as the sum of edge weights (i.e., Fisher’s *z* scores) attached to a node (Fornito et al., 2016). The results were visualized using the BrainNet Viewer toolbox (Xia et al., 2013).

### Correlation Between Network Connectivity and Behavioral Performance

Partial correlation analysis was conducted between the connectivity strength of the networks showing between-group differences and the performance of handwriting, reading and cognitive tests, controlling for age, Raven IQ and FD. The connectivity strength was defined as the average of connectivity weights (Fisher’s z scores) of all edges of the networks. The statistical significance was set at *p* < 0.05, uncorrected for the multiple comparisons.

### Validation Analysis

To evaluate the robustness of our results, two validation procedures were performed. First, we repeated the whole data analysis by using the FC matrices that were survived at a less stringent threshold of *p* < 0.05, uncorrected for multiple comparisons. Second, we reanalyzed the NBS analysis using NBS extent, in which the size of a network component is defined as the total number of connections it comprises.

## Results

### Behavioral Results

#### Out-Scanner Behavioral Performance

The results of reading, handwriting and cognitive skills tests are presented in Table 1. Results indicated that dyslexics compared to controls showed inferior performance in reading fluency, phonological awareness and orthographic awareness. In addition, dyslexics showed poorer handwriting fluency (both characters and digits) than controls. However, dyslexics and controls did not differ in handwriting speed and quality in the pen-and-paper copying task. Finally, we found that, compared to controls, dyslexics exhibited reduced phonological working memory span, but exhibited intact sustain attention ability.

#### In-Scanner Behavioral Performance

**FIGURE 1:**
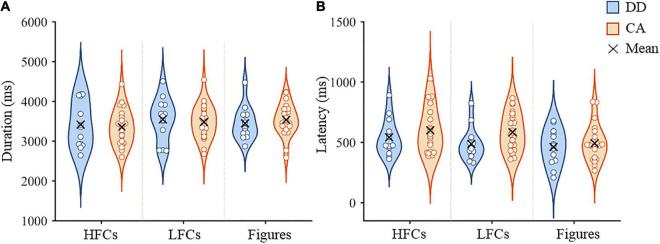
In-scanner performance in the copying and drawing tasks. Violin plots of writing duration **(A)** and writing latency **(B)** of dyslexic children and controls during copying HFCs and LFCs and drawing figures. HFCs = high-frequency characters, LFCs = low-frequency characters, DD = developmental dyslexia and CA = chronological-age matched control group.

The average writing duration and latency during fMRI scanning are presented in Figure 1. For writing duration, the interaction between group and stimulus type (*F*(2, 68) = 1.18, *p* = 0.314) and the main effect of group (*F*(1, 34) = 0.01, *p* = 0.930) were not significant. The main effect of condition was marginally significant (*F*(2,68) = 3.01, *p* = 0.056). *Post hoc* pairwise comparisons showed that the duration of copying HFCs was shorter than that of copying LFCs (*p* = 0.008), but there was no significant difference between copying HFCs and drawing figures (*p* = 0.119), or between copying LFCs and drawing figures (*p* = 0.658) (Figure 1A). For writing latency, the interaction between group and stimulus type (*F*(2, 68) = 0.80, *p* = 0.455) and the main effect of group (*F*(1,34) = 1.82, *p* = 0.186) were not significant. The main effect of condition was significant (*F*(2,68) = 6.23, *p* = 0.003). *Post hoc* pairwise comparisons showed that the latency of copying HFCs was longer than that of drawing figures (*p* = 0.001), but there was no significant difference between copying HFCs and copying LFCs (*p* = 0.126) and between copying LFCs and drawing figures (*p* = 0.064) (Figure 1B).

### Network-Based Statistic Analysis Results

**FIGURE 2:**
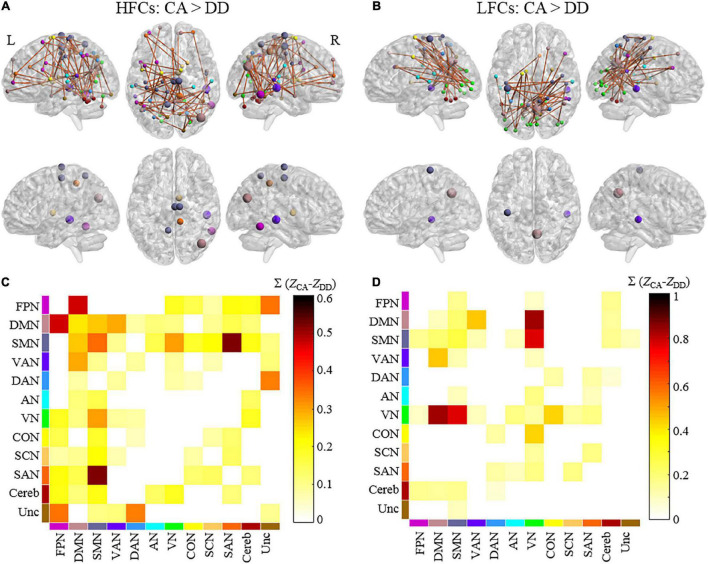
The differences in functional brain networks between dyslexics and controls during handwriting. Brain networks and hubs of the network showing stronger connectivity in controls than in dyslexics during copying HFCs **(A)** and LFCs **(B)**. The colors of the nodes indicate the network to which they belong. The size of hubs is proportional to their node strength. The matrix plots showing the connectivity patterns within/between each pair of networks during copying HFCs **(C)** and LFCs **(D)**. The color of each element in the matrices represents the sum of the differences in connectivity strength of all the edges for the connected networks. L = left and R = right. HFCs = high-frequency characters, LFCs = low-frequency characters, DD = developmental dyslexia, CA = chronological-age matched control group. FPN = frontal-parietal network, DMN = default mode network, SMN = somatomotor network, VAN = ventral attention network, DAN = dorsal attention network, AN = auditory network, VN = visual network, CON = cingulo-opercular network, SCN = subcortical network, SAN = salience network, Cereb = cerebellum and Unc = Uncertain.

The NBS analysis revealed that controls showed greater connectivity than dyslexic children in a functional brain network during copying HFCs, consisting of 66 nodes and 68 edges (Figure 2A). According to the functional network division (Power et al., 2011), this network can be grouped as internetwork connectivity between the sensory-motor network (SMN) and visual network (VN), between the default mode network (DMN) and ventral attention network (VAN), between the DMN and frontal-parietal network (FPN), and between the SMN and salience network (SAN) (Figure 2C and Supplementary Figure 1). Three nodes in the SMN (two nodes in the right medial frontal gyrus and the left precuneus), a node in the DMN (the right middle temporal gyrus), a node in the VAN (the right superior temporal gyrus) and a node in the FPN (the right inferior temporal gyrus) were identified as hubs (Supplementary Table 1).

During copying LFCs, controls also showed greater connectivity than dyslexic children in a functional brain network consisting of 48 nodes and 48 edges (Figure 2B). This network mainly encompassed internetwork connectivity between the SMN and VN, between the DMN and VAN, between the VN and DMN and between the VN and cingulo-opercular network (CON) (Figure 2D and Supplementary Figure 1). A node in the DMN (the right precuneus), a node in the VAN (the right superior temporal gurus) and a node in the SMN (the left precentral gyrus) were identified as hubs (Supplementary Table 1).

However, no significant differences in functional brain networks were detected between the two groups during drawing figures.

### Correlation Between Network Connectivity and Behavioral Performance

Correlation analysis revealed that connectivity strength of the functional networks showing group differences was positively correlated with writing speed of digits (HFCs: *r* = 0.37, *p* = 0.032; LFCs: *r* = 0.38, *p* = 0.027), reading fluency (HFCs: *r* = 0.53, *p* = 0.002; LFCs: *r* = 0.60, *p* < 0.001), orthographic awareness (ACC: HFCs: *r* = 0.30, *p* = 0.098; LFCs: *r* = 0.39, *p* = 0.026; RT: HFCs: *r* = -0.41, *p* = 0.021; LFCs: *r* = -0.39, *p* = 0.026) and phonological working memory (HFCs: *r* = 0.40, *p* = 0.023; LFCs: *r* = 0.37, *p* = 0.033).

### Validation Results

We found that when using a less stringent threshold for determining the FC metrics, dyslexics showed weaker functional connectivity than controls in a network involving in the VN, SMN, DMN, VAN and FPN in both the HFC and LFC conditions. This result was similar to the reported findings, despite the slight differences in connectivity strength (Supplementary Figure 2). Similarly, in the context of using an alternative NBS estimation approach, between-group differences were also identified in a similar functional network as the reported findings in the two handwriting conditions (Supplementary Figure 3). Collectively, these results indicated that the differences in functional networks between dyslexic and control children during copying HFCs and LFCs were largely reproducible.

## Discussion

Using a network analysis approach, the study identified the aberrant functional brain networks associated with the handwriting deficit in Chinese children with dyslexia. We found that dyslexics showed reduced functional connectivity in large-scale brain networks during handwriting involving the VN, SMN, DMN, VAN and FPN, suggesting that task-relevant sensor-motor networks and domain-general executive control networks convergently contribute to the handwriting deficit in DD. Moreover, we found that the between-group differences in functional networks varied between the high-frequency and low-frequency conditions, suggesting that dyslexics’ handwriting deficit was mediated by linguistical variables.

Behaviorally, we found that dyslexics showed reduced handwriting fluency relative to controls. This result is in line with previous research indicating that dyslexics showed impaired handwriting fluency (Arfe et al., 2020). However, dyslexics and controls showed no statistically significant differences in writing speed and quality in the pen-and-paper copying task. This result is inconsistent with previous findings showing reduced handwriting speed during copying tasks in dyslexics relative to controls (Lam et al., 2011; Meng et al., 2019). These findings suggest that the fluency task paradigm may be more sensitive to capture the insufficient automaticity of handwriting in dyslexics than the copying tasks in behavioral measures. Alternatively, because dyslexics showed the trend of decreasing handwriting speed and quality in the copying tasks, we speculated that the failure to reach statistical significances may be associated with the small sample size.

In line with our hypothesis, the brain network analysis revealed that dyslexics and controls differed in functional connectivity in a distributed brain network supporting visual, motoric and cognitive executive processes. Furthermore, behavioral recordings during fMRI scanning showed no differences in task-performance between the two groups of participants, excluding the possibility that the observed differences in functional brain networks are just derived from task difficulty. These findings suggest that the handwriting problem in dyslexics is not derived from a low-level perception and motor dysfunction, but instead from a failure of the integration of cognitive, sensory and motor systems. This argument is supported by the brain-behavior correlation analysis showing that the brain networks showing between-group differences are related to the skills of handwriting speed, reading and working memory.

Another critical finding of this study is that we did not observe between-group differences in the drawing condition, suggesting that the observed functional network abnormalities in dyslexics are specific to handwriting processing. Handwriting and drawing skills share several basic sensory and motor processes, which are supported by an overlapped brain circuit (Yuan and Brown, 2015). However, we found the brain basis specific to the handwriting deficit in DD, suggesting that the brain systems for handwriting and drawing have been dissociation in the middle age of children. This view is supported by a developmental study that reported that children around 10 years old have established the brain system of handwriting (Palmis et al., 2021).

The connectivity profiles of the networks showing group differences were characterized by referring a functional network template (Power et al., 2011). First, we found that dyslexics showed altered internetwork connectivity between the VN (including the nodes of the fusiform gyrus and lingual gyrus) and the SMN (including the nodes of the precentral gyrus and postcentral gyrus), irrespective of character frequency. This result is in accordance with previous findings of reduced brain activation in the visual-orthographic regions (Cao et al., 2018; Yang et al., 2022) and the visual perception regions (Yang et al., 2021) in Chinese dyslexic children. The VN is thought to support visual analysis of Chinese characters during handwriting (Wu et al., 2012; Cao Q. et al., 2013). Consistent with this interpretation, correlation analysis showed that network connectivity was positively correlated with orthographic awareness. Moreover, the SMN has been widely identified to be engaged in handwriting. Functionally, the bilateral primary motor regions are involved in motor control (Planton et al., 2013), while the medial frontal gyrus (including the SMA) serves the process of Chinese writing sequence (Zhang Z. et al., 2021) or motor response preparation (Planton et al., 2013). Consequently, the coupling between the SMN and VN is recruited to support the coordination of visual and motor controls necessary for handwriting. Alternatively, the SMN and VN may be recruited to serve the sensory feedbacks during handwriting, which plays an important role in optimizing motor output (Peterka, 2002). Because handwriting has not yet become fully automatic in children, an attentional controlled movement pattern is engaged in handwriting, which highly relies on visual and somatomotor feedbacks (Marquardt et al., 1999). Thus, the reduced connectivity between the VN and SMN may affect the functional integration engaged in the sensory feedback processing, thus slowing down handwriting speed or wrecking handwriting quality in dyslexics.

Another important finding of the present study is the disrupted connectivity of the DMN with multiple functional networks during handwriting in Chinese dyslexics. This result is consistent with the view that the DMN serves as an “integrative hub” for the cross talk between functional brain networks (Braga et al., 2013). First, the connectivity between the DMN and VAN was decreased in dyslexics relative to controls, which was observed in both high-frequency and low-frequency conditions. Although the altered connectivity of the DMN has been repeatedly identified in DD during resting and task states (Finn et al., 2014; Schurz et al., 2015), the specific role of the DMN in dyslexia is still unclear. In this sense, the identified association between the disruption of the DMN and handwriting deficit hints on a possible role of the DMN in dyslexia. The DMN is traditionally regarded as a task-negative functional network, whose activity is increased in internally oriented cognitive states (Raichle et al., 2001; Fox et al., 2005). According to this account of the DMN, dyslexia has been postulated to be associated with the failure of disengaging the DMN from reading-related networks (Boros et al., 2016; Cao et al., 2017). However, a growing body of empirical evidence suggests that the DMN is actively involved in goal-directed cognitive processing, such as task shift (Crittenden et al., 2015) and working memory (Spreng et al., 2014). Specifically, the DMN has been found to be involved in the application of learned information to make predictions in decision-making (Vatansever et al., 2017) and in the integration of external goals and internal representation (Spreng et al., 2014). Moreover, a recent study has demonstrated that the DMN encodes information associated with ongoing cognition for the memory-based guide in automated processing (Sormaz et al., 2018). Based on these findings, we posited that the DMN may play a role in high-level executive control for the integration of different brain systems involved in handwriting. In addition, the DMN may encodes the long-term representation of handwriting rules resulted from learning and practice. In line with this view, a prior study has demonstrated that the DMN is involved in visual-motor learning (Eryurek et al., 2022). On the other hand, the VAN is an attentional control network that serves the processing of unexpected stimuli, reflecting the bottom-up control processing, consistent with previous findings of the dysfunction of ventral and dorsal attentional networks in dyslexics (Meri et al., 2020). Previous studies have also found that the VAN was positively correlated with DMN during childhood (Barber et al., 2013). Thus, the disruption of the internetwork connectivity between the DMN and VAN may impede the cross talk between the internal representation of handwriting rules and external task contexts during handwriting in DD.

In addition to the common brain network abnormalities across the HFCs and LFCs conditions, we also found some differences in network connectivity between the two conditions. The frequency effect is a typical lexical variable that has been found to influence orthographic access during handwriting, and thus this result suggests the impact of linguistic factors to the handwriting deficit in DD. First, we found that the decreased connectivity between the DMN and the FPN in dyslexics was more evident in the high-frequency condition relative to the low-frequency condition. The FPN is a high-order control network for cognitive processes that flexibly interacts with other networks adapted to task demands (Cole et al., 2013). Coupled with the integrative role of the DMN in cognitive tasks (Braga et al., 2013; Wang et al., 2021), the internetwork connectivity between the FPN and DMN represents a neural circuit for executive control (Wang et al., 2021). Consistent with this interpretation, it has been reported that the connectivity between the FPN and DMN increases under the context of intentional speed-control processing in handwriting (Li et al., 2021). The linguistic information of the characters (phonologic or semantic) is more likely to be activated for the HFCs relative to LFCs, which may play an interferential role in the copying task because the orthographic forms have already been presented and thus the phonological or semantic information is not necessary. The disrupted connectivity between the FPN and DMN may hinder the adaptive control process during handwriting familiar HFCs in dyslexic children. In accordance with this explanation, functional connectivity between the DMN and executive control regions has been found to support the goal-directed semantic retrieval (Krieger-Redwood et al., 2016).

In addition, we found that dyslexics showed more pronounced abnormality in functional connectivity of the VN with the DMN and the CON in the LFCs condition. This result agrees with a resting-state functional connectivity study reporting the abnormalities of functional connectivity between the visual networks and prefrontal attention areas and the connectivity between the DMN and VN (Finn et al., 2014). The CON is a vital network hub of executive control that is thought to support the maintenance of task goals, the adjustments for feedback control and error monitor (Power et al., 2011). The reorganization of the CON has been found to be associated with reading improvement in DD (Horowitz-Kraus et al., 2015). Similarly, the DMN has been reported to generate top-down predictions by integrated memory-based information for automated cognitive processing (Vatansever et al., 2017). Thus, the reduced functional connectivity of the VN with the DMN and CON may reflect the problematic regulation from the DMN and CON to unimodal visual processing during handwriting in dyslexics. This explanation is favored by previous studies reporting the visual attention deficit in DD (Taran et al., 2022). Because the low-frequency characters were less familiar to the participants, the visual-orthographic processing may be more demanding in the LFCs condition relative to the HFCs condition, requiring more top-down control from the executive control regions to visual regions. Consequently, we found that the specific impairments in functional connectivity of the visual networks with the executive control networks during writing infrequent characters in dyslexics.

## Conclusion

Using a network analysis method, this study revealed that the handwriting deficit in DD was associated with the abnormalities of network connectivity in multiple brain networks involved in visual-orthographic, motor and executive control processes. Our findings advance our understanding of the brain basis of DD.

## Data Availability Statement

The data that support the findings of this study are available from the corresponding authors upon reasonable request.

## Ethics Statement

The study was approved by the Ethics Committee of the Institute of Psychology at the Chinese Academy of Sciences. Written informed consent to participate in this study was provided by the participants’ legal guardian/next of kin.

## Author Contributions

ZL: conceptualization, methodology, visualization, validation, formal analysis, investigation and writing—original draft. JL: methodology, investigation and formal analysis, and writing—original draft. H-YB: conceptualization, project administration and writing—review, and editing. MX: conceptualization, formal analysis, writing—review and editing, and supervision. YY: conceptualization, methodology, funding acquisition, resources, writing—review and editing, and supervision. All authors contributed to the article and approved the submitted version.

## Conflict of Interest

The authors declare that the research was conducted in the absence of any commercial or financial relationships that could be construed as a potential conflict of interest.

## Publisher’s Note

All claims expressed in this article are solely those of the authors and do not necessarily represent those of their affiliated organizations, or those of the publisher, the editors and the reviewers. Any product that may be evaluated in this article, or claim that may be made by its manufacturer, is not guaranteed or endorsed by the publisher.

## Footnotes

1
http://www.fil.ion.ucl.ac.uk/spm/
