# Supplementary material for: Disruption of Functional Brain Networks Underlies the Handwriting Deficit in Children With Developmental Dyslexia
Source: Front Neurosci. 2022 Jul 18;16:919440. doi: 10.3389/fnins.2022.919440 (PMC9339653; doi:10.3389/fnins.2022.919440)
Supplement: Supplementary file 1 [file Data_Sheet_1.pdf]

Table S1 Hubs in the functional brain networks showing group differences.

Brain region	Coordinates in Talairach space			Node strength	Network
	X	Y	Z		
Copying high-frequency characters: controls > dyslexics					
Middle temporal gyrus	39	-71	24	0.58	DMN
Superior temporal gyrus	47	-29	-2	0.52	VAN
Inferior temporal gyrus	53	-51	-12	0.50	FPN
Medial frontal gyrus	1	-22	54	0.45	SMN
Medial frontal gyrus	8	-23	69	0.43	SMN
Precuneus	-8	-55	54	0.42	SMN
Precuneus	9	-42	46	0.41	SAN
Thalamus	7	-6	9	0.40	SCN
Copying low-frequency characters: controls > dyslexics					
Precuneus	4	-59	31	0.62	DMN
Superior temporal gyrus	47	-29	-2	0.45	VAN
Precentral gyrus	-37	-32	63	0.44	SMN

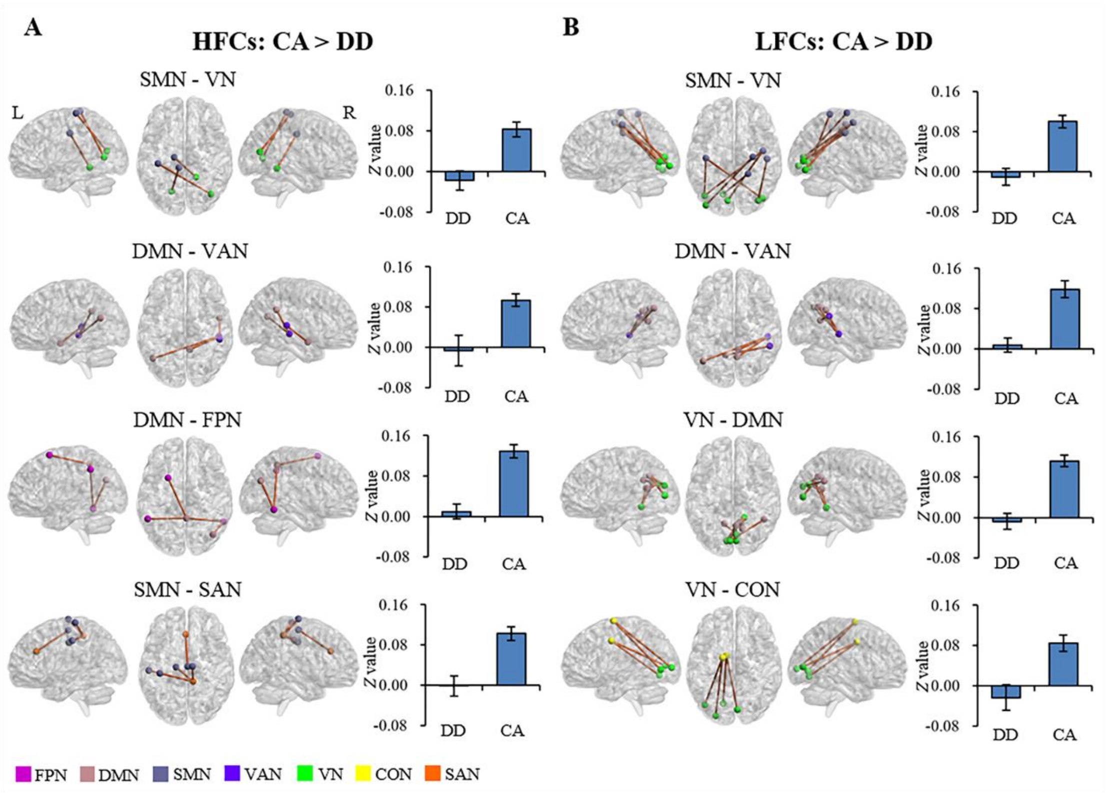

Figure S1 The internetwork connectivity profiles in the functional brain networks showing group differences (B). L = left and R = right. HFCs = copying high-frequency characters and LFCs = copying low-frequency characters. DD = developmental dyslexia, CA = age-matched ~~controls~~. FPN = frontal-parietal network, DMN = default mode network, SMN = somatomotor network, VAN = ventral attention network, VN = visual network, CON = cingulo-opercular network and SAN = salience network.

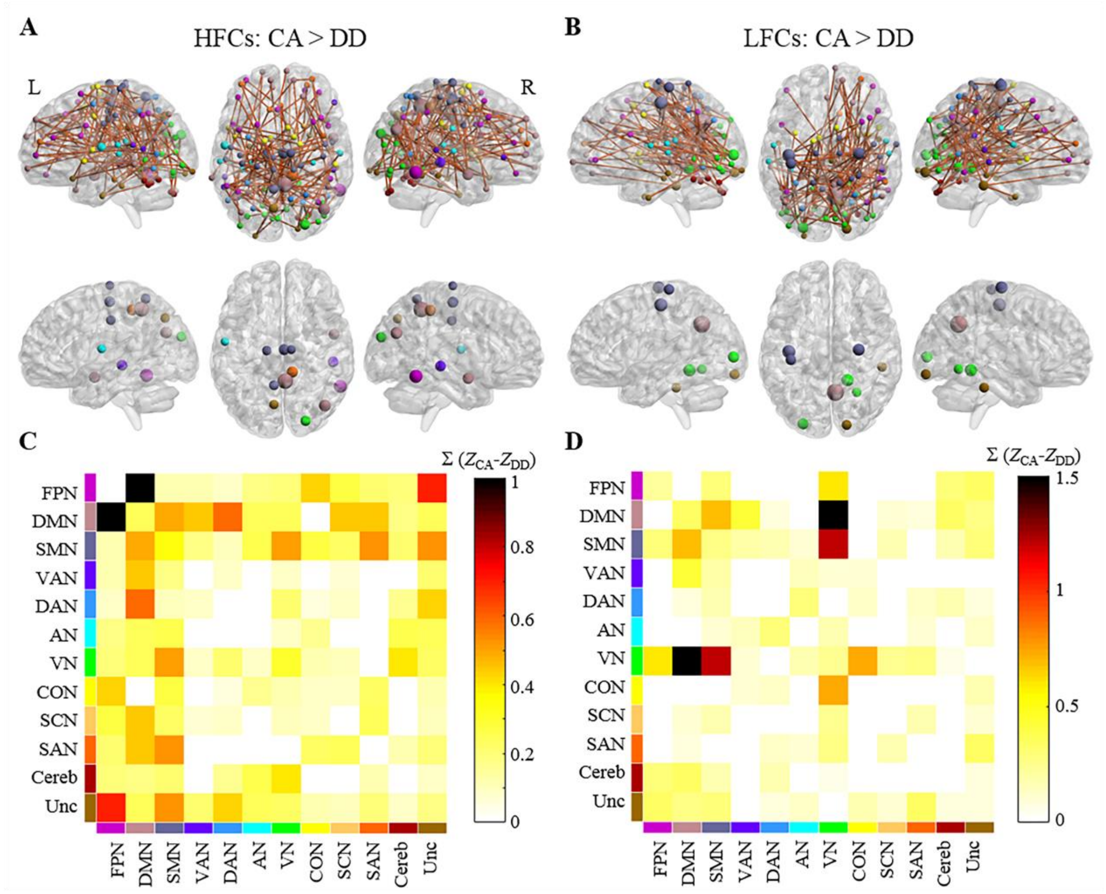

Figure S2 The results of functional brain networks based on the threshold of $p < 0.05$ for determining the FC metrics. Brain networks and hubs of the networks showing stronger connectivity in controls than in dyslexics during copying HFCs (A) and LFCs (B). The matrix plots show the connectivity patterns within/between each pair of networks in the HFCs (C) and LFCs (D) conditions. L = left and R = right. HFCs = high-frequency characters, LFCs = low-frequency characters, DD = developmental, dyslexia, CA = age-matched ~~controls~~. FPN = frontal-parietal network, DMN = default mode network, SMN = somatomotor network, VAN = ventral attention network, DAN = dorsal attention network, AN = auditory network, VN = visual network, CON = cingulo-opercular network, SCN = subcortical network, SAN = salience network, Cereb = cerebellum and Unc = Uncertain.

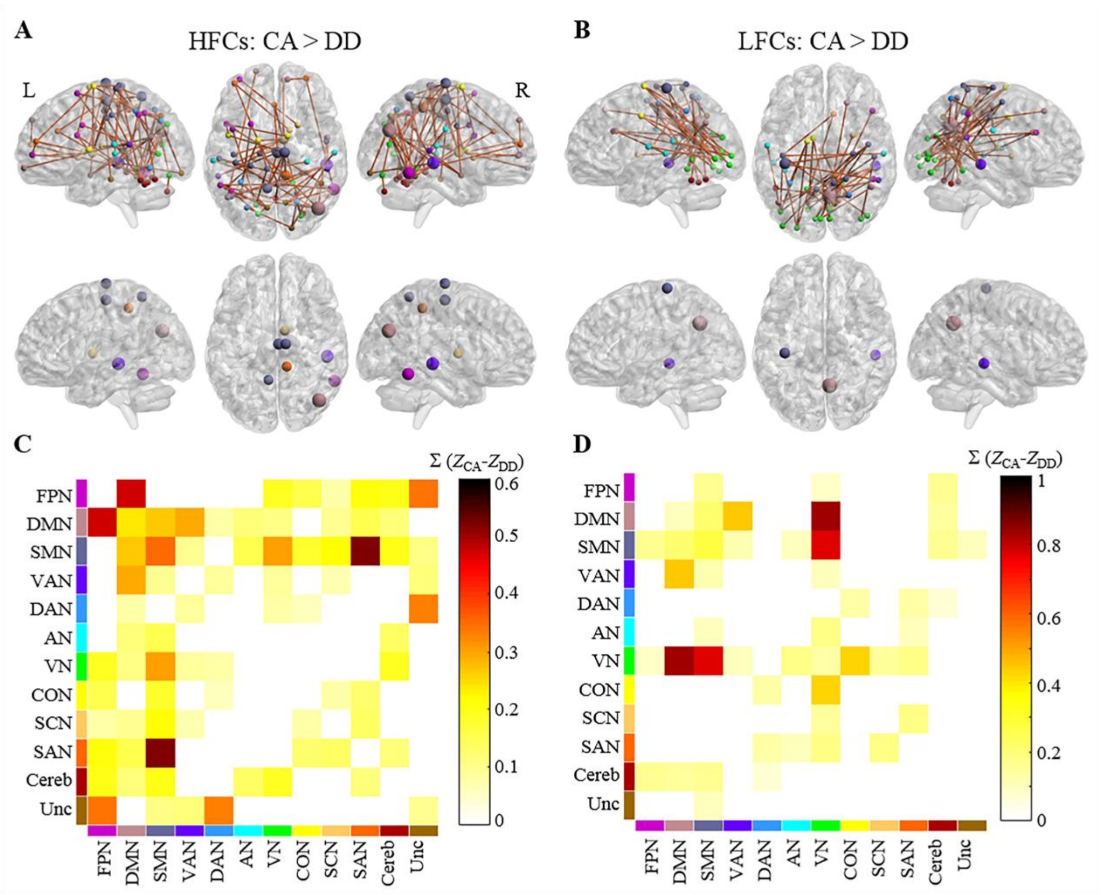

Figure S3 The results of functional brain networks for using NBS extent ~~in the nonparametric permutation test~~. Brain networks and hubs of the networks show stronger connectivity in controls than in dyslexics in the HFCs (A) and LFCs (B) conditions. The matrix plots show the connectivity patterns within/between each pair of networks in the HFCs (C) and LFCs (D) conditions. L = left and R = right. HFCs = high-frequency characters, LFCs = low-frequency characters, DD = developmental dyslexia, CA = age-matched ~~controls~~. FPN = frontal-parietal network, DMN = default mode network, SMN = somatomotor network, VAN = ventral attention network, DAN = dorsal attention network, AN = auditory network, VN = visual network, CON = cingulo-opercular network, SCN = subcortical network, SAN = salience network, Cereb = cerebellum and Unc = Uncertain.
